# Factors Influence the Acceptance of Surgical Treatment in Chinese Bariatric Surgery Candidates

**DOI:** 10.1007/s11695-018-3237-5

**Published:** 2018-04-29

**Authors:** Shutong Tang, Shuqing Yu, Cunchuan Wang, Jingge Yang, Lilian Gao, Xiaomei Chen, Lina Wu, Bingsheng Guan, Jinfen Han, Weiju Chen, Wah Yang

**Affiliations:** 1 Department of Nursing, The First Affiliated Hospital, Jinan University, Guangzhou, China; 2 Department of Metabolic and Bariatric Surgery, The First Affiliated Hospital, Jinan University, Guangzhou, China; 3 Joint Institute of Metabolic Medicine between State Key Laboratory of Pharmaceutical Biotechnology, The University of Hong Kong and Jinan University, Guangzhou, China; 4 State Key Laboratory of Pharmaceutical Biotechnology, LKS Faculty of Medicine, The University of Hong Kong, Hong Kong, China; 5 Department of Medicine, LKS Faculty of Medicine, The University of Hong Kong, Hong Kong, China

**Keywords:** Factors, Acceptance, Decision, Bariatric surgery, China

## Abstract

**Background:**

The prevalence of obesity and obesity-related disorders is rapidly increasing among the Chinese populations. Bariatric surgery is becoming more and more popular in China, yet little cases were performed compared with western countries. The acceptance of this new treatment modality in Chinese bariatric surgery candidates was seldom studied.

**Objective:**

To investigate the factors affecting the choice of bariatric surgery in Chinese patients with obesity and metabolic disorders, so as to promote the popularization of bariatric surgery in developing countries like China

**Methods:**

Patients with obesity and related metabolic disorders meet the indications for bariatric surgery in the Department of Metabolic and Bariatric Surgery in the First Affiliated Hospital of Jinan University between January 2016 and April 2017 were asked to answer a questionnaire about the demographics of the patients, social economic status, present and past history, family history, etc. The data collected and the relationship of the acceptance of bariatric surgery were analyzed.

**Results:**

There were 157 patients (51 males, 32.5%; 106 females, 67.5%) with mean BMI 38.7 ± 8.1 kg/m^2^ answered the questionnaire. One hundred twenty-three of them (78%) accepted bariatric surgery. By univariate analysis, it was found that patients’ weight, BMI, family support, medical insurance, past surgical history, family history of T2DM, and obesity-related comorbidities and symptoms are correlated with the acceptance of bariatric surgery. By multivariate analysis, it was found that patients’ weight (*P* = 0.024), BMI (*P* = 0.007), family support (*P* < 0.001), medical insurance (*P* < 0.001), past surgical history (*P* = 0.011), family history of T2DM (*P* = 0.020), and obesity-related comorbidities and symptoms (*P* = 0.030) are statistically significant and were positively correlated with the acceptance of bariatric surgery. Age, height, gender, history of smoking and alcohol consumption, family history of obesity, history of hypertension and T2DM, education level, and marital status were not statistically significant (*P* < 0.05).

**Conclusions:**

Patients with heavier weight, higher BMI, family support, medical insurance reimbursement, past surgical history, family history of T2DM, and obesity-related comorbidities and symptoms are more likely to consider bariatric surgery in Chinese bariatric surgery candidates. It will be important to provide appropriate healthcare education and support to patients focusing on both obesity-related health risks and options of surgical treatment so to improve their acceptance of bariatric surgery.

The prevalence of obesity and obesity-related disorders is rapidly increasing among the Chinese population [1]. Bariatric surgery is becoming more and more popular in China [2], yet little cases were performed compared with western countries. The public and even physicians and other health-related professions may not know much about the bariatric surgery. The acceptance of this new treatment modality in Chinese bariatric surgery candidates was seldom studied.

## Methods

### Patients

Patients with obesity and related metabolic disorders meet the indications for bariatric surgery in the Department of Metabolic and Bariatric Surgery in the First Affiliated Hospital of Jinan University between January 2016 and April 2017 were asked to answer a questionnaire (Appendix I) about the demographics of the patients, social economic status, present and past history, family history, etc. All the patients answered the questionnaire. In this study, acceptance of surgery is defined as actual delivery of the surgical care that the patients underwent bariatric surgery. The patients accepted bariatric surgery were in surgical group while those not accept were in non-surgical group (reference group). The data collected and the relationship of the acceptance of bariatric surgery were analyzed. All of these patients met the minimal criteria for bariatric surgery proposed by the IFSO-APC Consensus Statements 2011 [3].

### Statistical Analysis

All clinical data were analyzed by SPSS (version 13.0) statistical software (SPSS Inc., Chicago, IL). Demographics, social status, and past history of the patients were analyzed by *χ*2 test for patients in surgical group and non-surgical group. Odds ratio (OR) and 95% CI were calculated by using the multivariate logistic regression model based on the univariate analyses. A *P* value < 0.05 was considered statistically significant.

## Results

There were 157 patients (51 males, 32.5%; 106 females, 67.5%) with mean BMI 38.7 ± 8.1 kg/m^2^ answered the questionnaire. One hundred twenty-three of them (78%) accepted bariatric surgery. Demographics of the patients are shown in Table 1.

**Table 1:** Demographics of the patients

	Surgical group *n* = 123		Non-surgical group *n* = 34		*P*
	*n*	%	*n*	%	
Gender					0.419
Male	38	30.9	13	38.2	
Female	85	69.1	21	61.8	
BMI (kg/m^2^)					0.028*
28–29.9	10	8.1	10	29.4	
30–34.9	27	22	10	29.4	
35–39.9	37	30.1	6	17.6	
40–49.9	37	30.1	7	20.7	
50–59.9	9	7.3	1	2.9	
≥ 60	3	2.4	0	0	
Age					1.000
≤ 17	9	7.4	2	5.9	
18–40	88	71.5	25	73.5	
41–65	26	21.1	7	20.6	

By univariate analysis, it was found that patients’ weight, BMI, family support, medical insurance, past surgical history, family history of T2DM, and obesity-related comorbidities and symptoms are correlated with the acceptance of bariatric surgery (Table 2).

**Table 2:** Social status and past history of the patients

	Surgical group *n* = 123		Non-surgical group *n* = 34		*P*
	*n*	%	*n*	%	
Social economic status					
Education					0.077
Illiteracy	1	0.8	2	5.9	
Primary school	7	5.7	3	8.9	
Middle school	23	18.7	6	17.6	
High school	20	16.3	1	2.9	
University/above	72	58.5	22	64.7	
Marriage					0.902
Single	59	47.9	17	50	
Married	56	45.5	16	47	
Divorced/widowed	8	6.5	1	3	
Medical insurance coverage					< 0.001*
Yes	105	85.4	9	26.5	
No	18	14.6	25	73.6	
Family support					< 0.001*
Yes	107	94.7	16	51.6	
No	6	5.3	15	48.4	
Present and past history, family history, obesity-related comorbidities					
Past surgical history					0.011*
Yes	55	44.7	7	20.6	
No	68	55.3	27	79.4	
Smoking					0.266
Yes	29	23.6	5	14.7	
No	94	76.4	29	85.3	
Alcohol consumption					0.910
Yes	14	11.4	3	8.8	
No	109	88.6	31	91.2	
Family history of obesity					0.158
Yes	71	57.7	15	44.1	
No	52	42.3	19	55.9	
Family history of T2DM					0.044*
Yes	60	48.8	10	29.4	
No	63	51.2	24	70.6	
History of hypertension					0.404
Yes	26	21.1	5	14.7	
No	97	78.9	29	85.3	
History of T2DM					0.343
Yes	23	18.7	4	11.8	
No	100	81.3	30	18.7	
Obesity-related comorbidities					0.018*
Yes	116	94.3	27	79.4	
No	7	5.7	7	20.6	

*P < 0.05, statistically significant

By multivariate analysis, it was found that patients’ weight (*P* = 0.024), BMI (*P* = 0.007), family support (*P* < 0.001), medical insurance (*P* < 0.001), past surgical history (*P* = 0.011), family history of T2DM (*P* = 0.020), and obesity-related comorbidities and symptoms (*P* = 0.030) are statistically significant and were positively correlated with the acceptance of bariatric surgery (Table 3). Age, height, gender, history of smoking and alcohol consumption, family history of obesity, past history of hypertension and T2DM, education level, and marital status were not statistically significant (*P* < 0.05).

**Table 3:** Multivariate analysis of acceptance of surgical treatment

Independent variables	OR	95%CI
Weight, kg	1.02	1.003–1.037
BMI, kg/m^2^	1.092	1.024–1.163
Family support	6.752	2.317–19.681
Medical insurance coverage	17.839	6.551–48.574
Past surgical history	3.12	1.264–7.705
Obesity-related symptoms and comorbidities	6.783	1.947–23.635
Family history of T2DM	2.867	1.183–6.950

*BMI* body mass index, *T2DM* type 2 diabetes mellitus

## Discussion

Bariatric surgery is becoming more and more popular in China as the number of obese patients in mainland China increased a lot in the past few decades [2]. Yet the development of bariatric surgery in mainland China is still in its initial stages, the public and even physicians and other health-related professions may not know much about the bariatric surgery. The acceptance of this new treatment modality in Chinese bariatric surgery candidates was seldom studied. Therefore, factors influence the acceptance of surgical treatment in Chinese bariatric surgery candidates were investigated in this study.

### Weight and BMI

Our findings revealed that weight and BMI of the patients are the factors affecting the acceptance of bariatric surgery while gender, age, and height do not. This finding is different from a study in the USA which showed that females were more likely to obtain bariatric surgery than males, and patients aged 40–59 years were more likely than other age groups to obtain bariatric surgery [4].

### Medical Insurance Coverage

China’s medical insurance system consists of government-run basic health insurance and commercial health insurance as described by Fang K. et al. [5]. There are three types of basic health insurance schemes for different groups of people in rural and urban areas, which are administrated by the central and local governments. In rural area, it is named as the new rural cooperative medical care system (NCMS). In urban area, it is named as the urban employee basic medical insurance for urban employed (UEBMI) and urban resident basic medical insurance for urban residents (URBMI). Although these three basic medical insurances cover almost all Chinese residents and up to 75% of medical expenses can be reimbursed, however, it does not cover all medical expenses [5]. Commercial health insurance is supplementary to basic health insurance, targeting mainly the upper class but only accounted for about 5.72% of all insurance reimbursements [6].

Medical expenditure (including medication, physical examinations and laboratory investigations, a variety of surgical disposable supplies) in China is divided into three levels (A, B, and C) by the government: Class A expenditures can be fully reimbursed, Class B reimbursable ratio ranging from 5 to 30% in accordance with the different provincial policies, Class C is a completely self-financed project. As a result, a large part of the self-pay has been generated; it is also named as out-of-pocket (OOP) payments. In 1998, the OOP payments as a share of total health expenditures and disposable personal income roared up to 55 and 5% due to the shrinkage of health insurance coverage [7]. Although since then, the Chinese governments have begun to solve that problem by implementing a series of reforms on the healthcare system, but it is still a problem that high levels of public concern and wait be solve until now.

For bariatric surgery, there are some surgical instruments and some other items belong to the range of OOP; more importantly, simple obesity is not included in the scope of China’s health insurance. Only in the case of other diseases such as metabolic syndromes and T2DM will be reimbursed at certain percentage by the government insurance. In some provinces of China, bariatric surgery is not covered by the medical insurance. And some of the patients do not even have other commercial insurance to cover the surgical expenses. Therefore, these patients need to pay on their own for bariatric surgery.

In this study, patients with medical insurance coverage got a higher rate of acceptance of the bariatric surgery than those without insurance coverage (Table 2). This showed that medical insurance coverage is one of the important factors affecting the acceptance of bariatric surgery, which is consistent with the current insurance policy on bariatric surgery. The local authorities may consider and provide more insurance coverage for the patients who have indications for bariatric surgery, especially for those with low incomes.

### Family Support

Our data showed that family support is positively correlated to the acceptance of the bariatric surgery. There were 12 (8%) patients did the surgery without telling their family. And eight (5%) patients insist of having surgery even opposed by their family. A study showed that people worry about the bariatric surgery is too risky even it is proved safe and effective [8]. Thus, we suggest that raising the knowledge and perception of the public to bariatric surgery is necessary.

### Obesity-Related Symptoms and Comorbidities

In our study, the patients with obesity-related symptoms and comorbidities were more likely to accept bariatric surgery (Tables 2 and 4). Most of the symptoms and comorbidities directly affect the safety and quality of life, such as snoring (67.5%), knee joints pain (39.8%), and shortness of breath (38.9%). These may be the main reasons for the patients seeking for surgical treatment. The result is similar with other study that treatment for medical problems is the primary reason seeking for bariatric surgery [9].

**Table 4:** Obesity-related symptoms and comorbidities

	Surgical group		Non-surgical group *n* = 34		Total *n* = 157	
	*n*	%	*n*	%	*n*	%
Shortness of breath	52	42.2	9	26.5	61	38.9
Insomnia	26	21.1	6	17.6	32	20.4
Headache in morning	21	17.0	3	8.8	24	15.3
Sense of hunger	23	18.7	5	15.7	28	17.8
Knee joints pain	53	43.0	9	26.5	62	39.5
Snoring	85	69.1	21	61.8	106	67.5
Sleep apnea	24	19.5	4	11.8	28	17.8
Somnolence	32	26.0	5	15.7	37	23.6
Reflux	31	25.2	5	15.7	36	22.9
Difficulty in pregnancy	10	8.1	0	0	10	6.4
No any	7	5.7	7	20.6	14	8.9

Our samples also found that past surgical history and family history of T2DM were positively correlated with the acceptance of bariatric surgery (Tables 2 and 3). Seventy of 157 cases (44.6%) had family history of T2DM. With these medical histories, the patients and their family members may concern more about their health condition and may proceed to more positive treatment modalities. However, the personal history of T2DM and history of hypertension were not statistically significant in this cohort, which is different from some study that the risk of T2DM is one of the reasons for seeking surgery [9]. Most of the Chinese patients with T2DM are treated by medication rather than surgery. In China, bariatric surgery is still new to the public even to the healthcare professions. The awareness of bariatric surgery is low. Therefore, medical treatment is still the first choice for the T2DM patients. And the more important thing is that the patients with family history of T2DM may care more about their health issues. This may explain that although family history of T2DM was a factor that contributed for acceptance of bariatric treatment, the actual presence of the disease was not.

### Others: Smoking, Alcohol Consumption, Education, and Marriage

Our study also revealed that smoking, alcohol consumption, education, and marriage status were not statistically significant in relation of acceptance of bariatric surgery. But we found that 34 of 157 (21.7%) patients had smoking history. Study showed that smoking is one of the risk factors that cause postoperative complications [10]. So, anti-smoking education should be conducted to the patients before and after surgery. A study from the USA showed that patients with private insurance may affect the educational resource utilization that they may obtain more educational information [11]. Besides, a study showed that education level of the patients is positively correlated with the acceptance of bariatric surgery [12] which is different from our samples.

### Sources of Information about Bariatric Surgery

Sources of information about bariatric surgery the patient obtained were also investigated in this study (Table 5). Most of the patient got the information through the Internet (43.3%) and mobile phone messenger APPs (15.9%). Another important source is based on the information given by friends and relatives (28.0%). On the contrary, the traditional media such as the newspaper only take small portion (9.6%). This revealed that the new technology media and peer influence are worthy of attention in China. We suggest that the promotion and education on bariatric surgery to the public should be focused more on these media.

**Table 5:** Sources of information about bariatric surgery

	Surgical group *n* = 123		Non-surgical group *n* = 34		Total *n* = 157	
	*n*	%	*n*	%	*n*	%
Internet	57	46.3	11	32.4	68	43.3
Newspaper	12	9.7	3	8.8	15	9.6
Mobile messenger APPs	22	17.9	3	8.8	25	15.9
Friends and relatives	33	26.8	11	32.4	44	28.0
Other	13	10.6	9	26.5	22	14.0

## Limitations

The study only investigated the pre-operation data. Post-operation weight loss outcomes and psychological data can be further analyzed in the future to see any relationship between two of these. There may be also several potential sources of bias in this study design of voluntary self-reported data.

## Conclusions

Patients with heavier weight, higher BMI, family support, medical insurance reimbursement, past surgical history, family history of T2DM, and obesity-related comorbidities and symptoms are more likely to consider bariatric surgery in Chinese bariatric surgery candidates. It will be important to provide appropriate healthcare education and support to patients focusing on both obesity-related health risks and options of surgical treatment so to improve their acceptance of bariatric surgery.
